# Spatial distribution and identification of potential risk regions to rice blast disease in different rice ecosystems of Karnataka

**DOI:** 10.1038/s41598-022-11453-9

**Published:** 2022-05-06

**Authors:** Chittaragi Amoghavarsha, Devanna Pramesh, Shankarappa Sridhara, Balanagouda Patil, Sandip Shil, Ganesha R. Naik, Manjunath K. Naik, Shadi Shokralla, Ahmed M. El-Sabrout, Eman A. Mahmoud, Hosam O. Elansary, Anusha Nayak, Muthukapalli K. Prasannakumar

**Affiliations:** 1 Department of Plant Pathology, University of Agricultural and Horticultural Sciences, Shivamogga, Karnataka India; 2 Rice Pathology Laboratory, All India Coordinated Rice Improvement Programme, University of Agricultural Sciences, Raichur, Karnataka India; 3 Center for Climate Resilient Agriculture, University of Agricultural and Horticultural Sciences, Shivamogga, Karnataka India; 4 Division of Social Sciences, Research Centre, ICAR-Central Plantation Crops Research Institute, Mohitnagar, Jalpaiguri, West Bengal India; 5 Centre for Biodiversity Genomics, University of Guelph, Guelph, ON N1G 2W1 Canada; 6 Department of Applied Entomology and Zoology, Faculty of Agriculture (El-Shatby), Alexandria University, Alexandria, 21545 Egypt; 7 Department of Food Industries, Faculty of Agriculture, Damietta University, Damietta, Egypt; 8 Plant Production Department, College of Food and Agriculture Sciences, King Saud University, Riyadh, 11451 Saudi Arabia; 9 Department of Plant Pathology, College of Agriculture, GKVK, University of Agricultural Sciences, Bengaluru, Karnataka India

**Keywords:** Ecology, Plant sciences

## Abstract

Rice is a globally important crop and highly vulnerable to rice blast disease (RBD). We studied the spatial distribution of RBD by considering the 2-year exploratory data from 120 sampling sites over varied rice ecosystems of Karnataka, India. Point pattern and surface interpolation analyses were performed to identify the spatial distribution of RBD. The spatial clusters of RBD were generated by spatial autocorrelation and Ripley’s K function. Further, inverse distance weighting (IDW), ordinary kriging (OK), and indicator kriging (IK) approaches were utilized to generate spatial maps by predicting the values at unvisited locations using neighboring observations. Hierarchical cluster analysis using the average linkage method identified two main clusters of RBD severity. From the Local Moran’s I, most of the districts were clustered together (at I > 0), except the coastal and interior districts (at I < 0). Positive spatial dependency was observed in the Coastal, Hilly, Bhadra, and Upper Krishna Project ecosystems (p > 0.05), while Tungabhadra and Kaveri ecosystem districts were clustered together at p < 0.05. From the kriging, Hilly ecosystem, middle and southern parts of Karnataka were found vulnerable to RBD. This is the first intensive study in India on understanding the spatial distribution of RBD using geostatistical approaches, and the findings from this study help in setting up ecosystem-specific management strategies against RBD.

## Introduction

Rice (*Oryza* *sativa* L.) is the most widely consumed cereal staple food which provides livelihood and nutritional security to the world’s population^1^. Owing to its significance, rice has been placed as a second important crop, yet it serves as an essential food source for Asian countries, particularly in South-Eastern parts^2^. Being a tropical and subtropical crop, it is highly adaptable to diverse growing environments and cropping conditions. The recognized rice ecosystems are irrigated lowland, rainfed lowland, upland, and flood-prone^3^. The most commonly followed method in a major portion of the world involves submerging in water, the most flourishable rice-growing system^4^. The topmost constraint for increasing rice production is the rice blast disease (RBD) caused by an Ascomycete fungus *Magnaporthe* *oryzae* Couch (syn. *Pyricularia* *oryzae* Cavara), which results in 10–30 percent annual yield loss in different production zones every year^5^, and up to 80–100 per cent losses under substantial epidemics^6^. The pathogen directly decreases rice yields and indirectly increases production costs^7^

Up-to-date RBD is of serious concern to the farming community due to its nature of survival, fast-spreading, and concurrent occurrences to traditional and non-traditional growing belts^8^. The pathogen produces numerous air-borne conidia, dispersing to kilometers depending on wind velocity^9^. To explicit spatial distribution of the disease, spatial pattern analysis of RBD is a suggestive approach in finding clues for disease epidemiology^8^. The spatial pattern of disease reflects the pattern of primary inoculum, mechanism of dispersal, a clue of inoculum source, means of dispersal, and the critical factors governing disease epidemics that help refine the strategies of disease monitoring and management^8,10,11^.

Many novel approaches have been utilized to describe the spatial pattern analysis of plant diseases^12–14^. Among them, geostatistical techniques are widely used to characterize the spatial patterns of plant diseases and to identify the potential risk factors involved in epidemics^15–18^. In the recent past, geographical information system (GIS) offers a platform to integrate geographical information, plant disease status, and meteorological data into one system, thereby enabling the relationship between plant disease progress and the environment^19^. Using GIS, the spatial positions of the pathogens and the disease-affected fields can be characterized^8^. With the GIS, geostatistical and hot spot analysis, interpolation, interpretation of semivariograms, and another modeling can be made to understand the progress of plant diseases over time and space^15,20^.

Spatial autocorrelation can be performed to determine the correlation between spatial data at varying intervals to find the spatial dependence^21,22^. With the use of an array of observations in space, models of spatial dependence can be expressed as semivariograms, through which the occurrence of disease with least variance and without bias can be estimated by kriging interpolation techniques^8,23,24^.

The Karnataka state is one of the major rice producers in India, and it has varied geographical pattern and ecosystems. In Karnataka, rice is mainly grown in different ecosystems like an irrigated and rainfed ecosystem. Under irrigated ecosystems, there are five major ecosystems viz., Bhadra, Kaveri, Tunga Bhadra Project (TBP), Upper Krishna Project (UKP), and Varada command area. The area under these ecosystems has a major share in paddy cultivation concerning area and production. The regions coming under rainfed ecosystems are classified into hilly and coastal ecosystems. The hilly ecosystem majorly consists of the districts of Western Ghats, whereas the coastal ecosystem consists of the districts alongside the Arabian Sea and called Karavali or Canara or Karnataka coast. Heavy rainfall pattern is the characteristic feature of hilly and coastal ecosystems^25,26^. Each ecosystem is unique concerning the availability of water, soil type, cultivars grown, etc. Each of these ecosystems has always suffered due to the occurrence of RBD in each season every year at severe form.

The previous workers made several attempts to document the disease status of rice blasts in Karnataka^27–29^, but the reports were limited to a particular ecosystem, and the information on disease spread covered all the ecosystems of the state is lacking. By considering the above grey areas, the present investigation aimed to determine the status and spatial distribution of RBD in diverse rice ecosystems of Karnataka, identify the hotspots/ clusters by employing point analysis, and estimate the potential risk areas of RBD in Karnataka using the interpolation technique.

## Results

### RBD severity in different rice ecosystems of Karnataka

**Figure 1:**
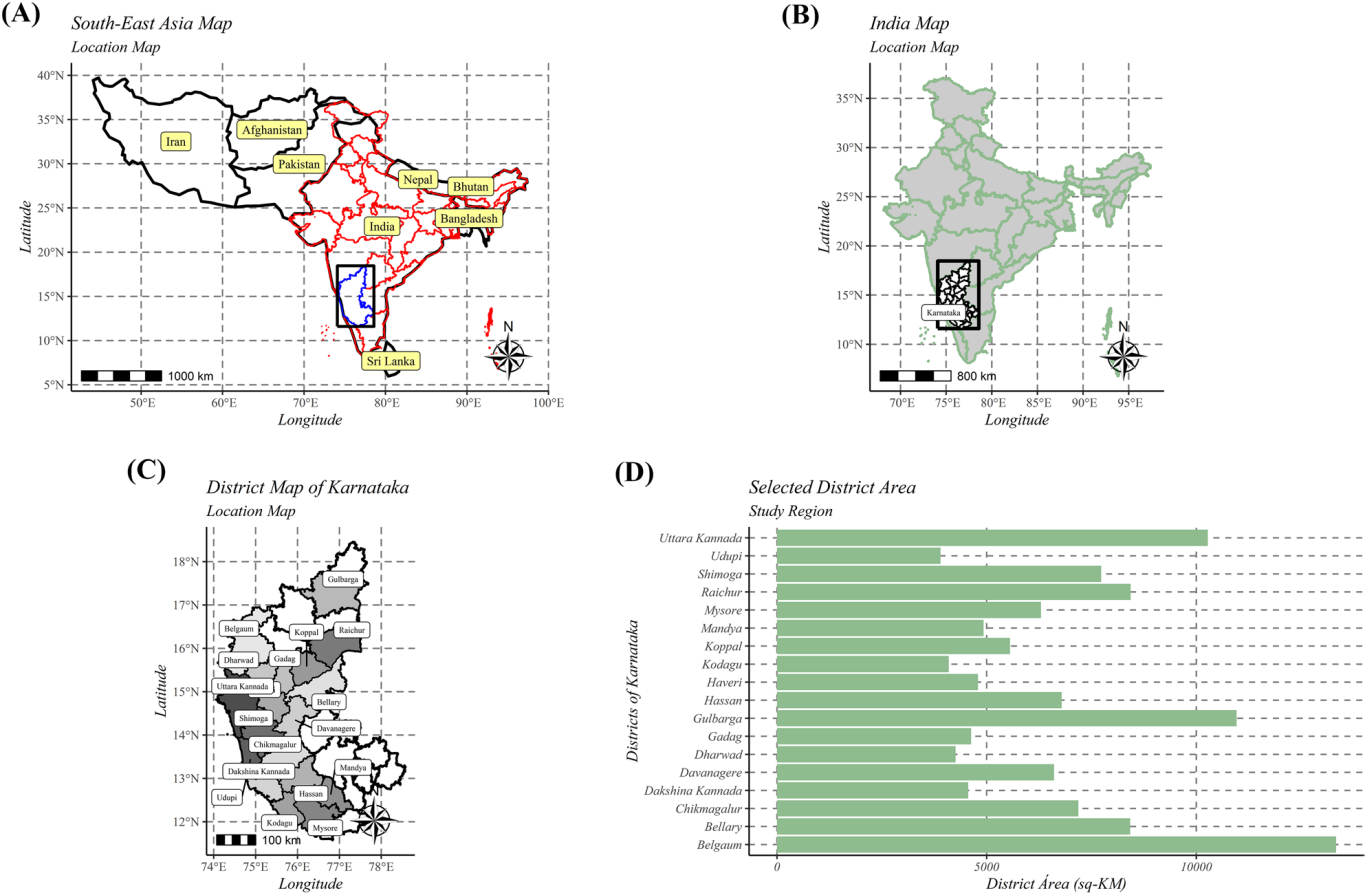
Featured map of South-East Asia (**A**), India (**B**), and Karnataka (**C**). A total of 18 administrative districts of Karnataka were considered to gather data on rice blast disease. The area of different districts under study is shown (**D**). The maps were created using R software (version R-4.0.3).

**Figure 2:**
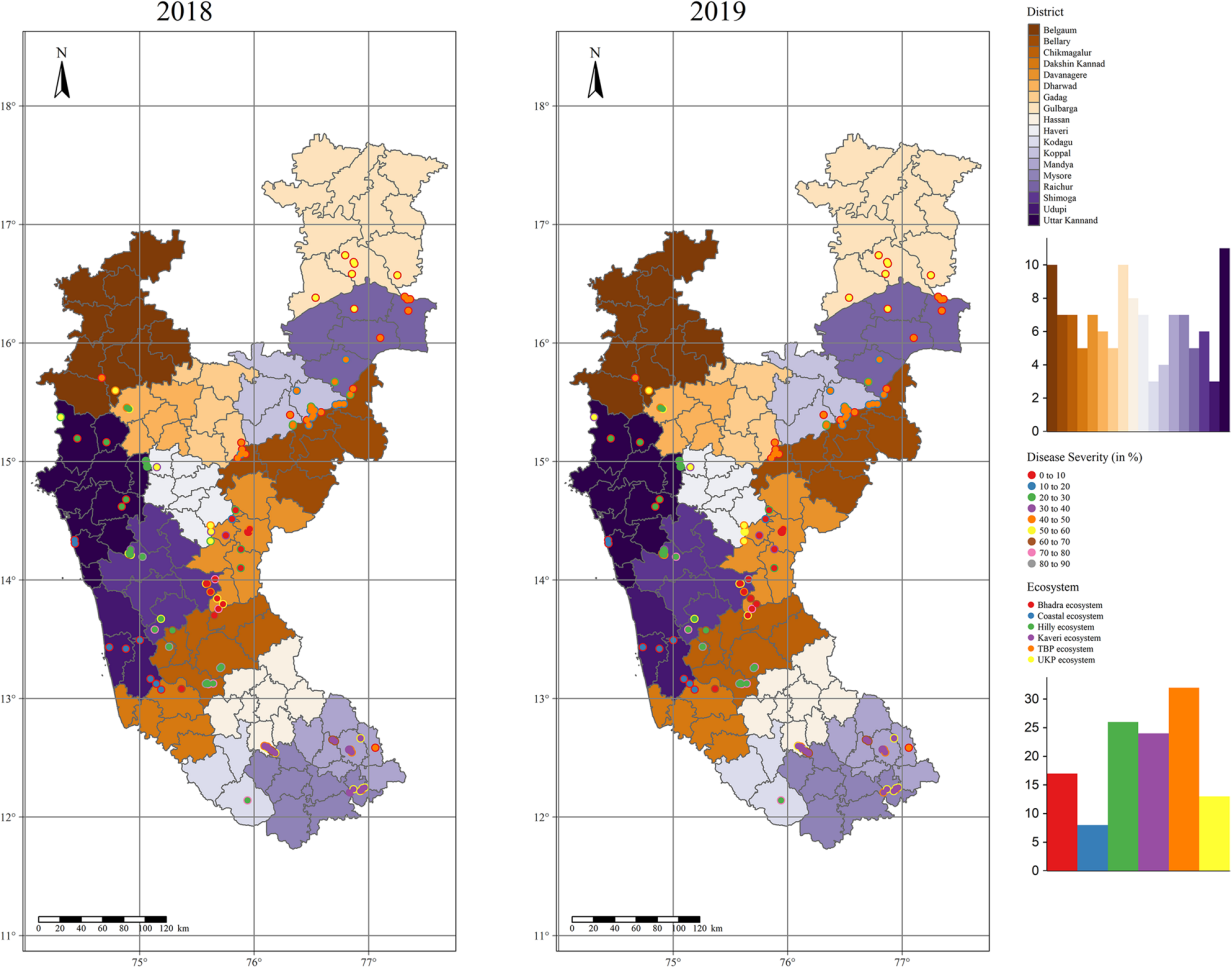
Distribution map indicating the sampling sites and the severity of rice blast disease in different rice ecosystems of Karnataka during 2018 and 2019. The maps were created using R software (version R-4.0.3).

**Figure 3:**
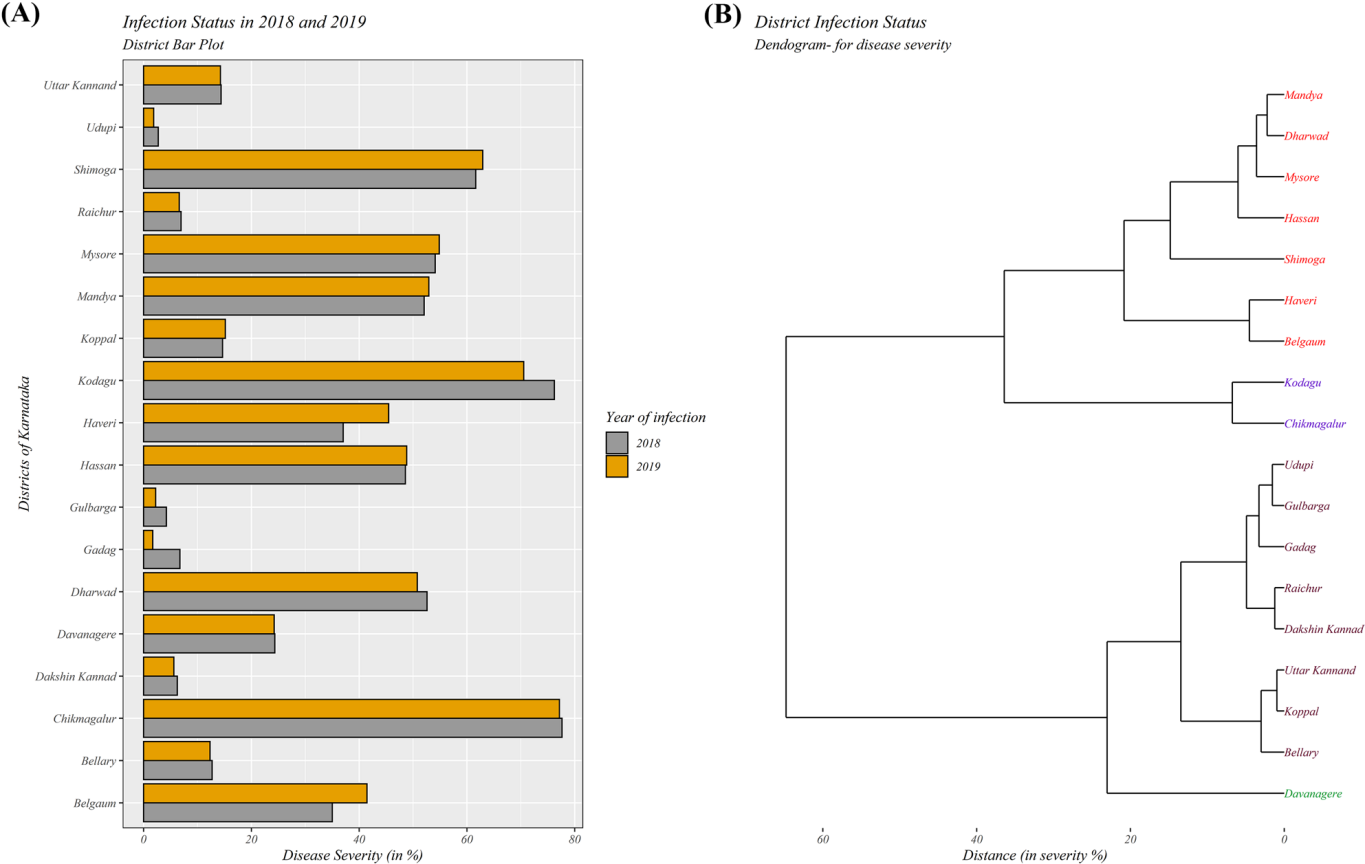
(**A**) Bar graph repressing the severity of rice blast disease (RBD) in different districts of Karnataka during 2018 and 2019. (**B**) Clustering of districts based on the severity of RBD in different districts of Karnataka by hclust method.

Based on the observations made during the exploratory surveys of 2018 and 2019 (Table 1 and Fig. 1), it was found that RBD severity significantly varied across studied areas and districts (Fig. 2). The disease severity was highest in Chikmagalur, followed by Kodagu, Shivamogga, Mysore, and Mandya districts which belong to Hilly and Kaveri ecosystems. At the same time, the lowest severity was documented in Udupi, Gulbarga, Gadag, Dakshin Kannad, Raichur, and Bellary districts of coastal, UKP, and TBP ecosystems (Fig. 3A).

**Table 1:** Details of diverse rice-growing ecosystems selected for the study.

Ecosystem	Districts	Agroclimatic zone	Rice cultivars
Tungabhadra project (TBP)	Bellary	Zone 3 (Northern Dry Zone)	BPT-5204, Gangavathi Sona, Kaveri Sona, GNV-10-89, RNR-15048, Nandhyal Sona and Nellur Sona
	Koppal	Zone 3 (Northern Dry Zone)	
	Raichur	Zone 2 (North Eastern Dry Zone) and Zone 3 (Northern Dry Zone)	
	Gadag	Zone 3 (Northern Dry Zone)	
Upper Krishna Project (UKP)	Gulbarga	Zone 3 (Northern Dry Zone)	BPT-5204, RNR-15048, Madhu and Sona
	Belgaum	Zone 3 (Northern Dry Zone) and Zone 8 (Northern Transition Zone)	Mangala, Intan, Belagavi Basmati, Kali kumad and Kumad
Varada command area	Haveri	Zone 8 (Northern Transition Zone)	Jyothi, IR-64, Rasi, Jaya and MTU 1010
Bhadra ecosystem	Shivamogga	Zone 7 (Southern Transition Zone)	Jyothi, Kempu Jyothi, Supriya Hybrid, JGL-1598, BPT-5204, Intan, RNR-15048, Mangala, Madhu, IR-20, Sharavathi, Aman Sona, Jeera and Nallur Sona
	Chikmagalur	Zone 4 (Central Dry Zone), Zone 7 (Southern Transition Zone)	
	Davanagere	Zone 3 (Northern Dry Zone), Zone 4 (Central Dry Zone) and Zone 7 (Southern Transition Zone)	
Kaveri ecosystem	Mysore	Zone 6 (Southern Dry Zone)	Jyothi, BR2655, Intan, Rajamudi, MC 13, MTU 1010, IR-64, CO-39, Thanu, Jaya, KRH-2 and KRH-4
	Mandya	Zone 6 (Southern Dry Zone)	
	Hassan	Zone 7 (Southern Transition Zone)	
Hilly ecosystem	Uttar Kannad	Zone 9 (Hill Zone)	Dodiga, Abhilash, Intan, Tunga, Jaya Navalisali, Neermulka, Bili Kagga, Mysuru Mallige, Jyothi, IR-64, MTU 1010, Rasi, Mangala, MTU 1001, KHP-2, IET7564 and IET-13549
	Dharwad	Zone 9 (Hill Zone)	
	Shivamogga	Zone 9 (Hill Zone)	
	Chikmagalur	Zone 9 (Hill Zone)	
	Kodagu	Zone 9 (Hill Zone)	
Coastal ecosystem	Dakshin Kannad	Zone 10 (Coastal Zone)	Kayame, Athikaya, Athikaraya, Hallaga, Kari Kagga, MO4, M021, Gandasali, Dodiga, Navalisali, Neermulka, Bili Kagga, Mysuru Mallige, Jyothi and IR-64
	Uttar Kannad	Zone 10 (Coastal Zone)	
	Udupi	Zone 10 (Coastal Zone)

Hierarchical cluster analysis using the average linkage method for RBD severity among the 18 administrative districts of diverse rice ecosystems of Karnataka identified two main clusters, namely, cluster I and cluster II (Fig. 3B). Cluster I consist of two subclusters, cluster IA and IB. Subcluster IA consists of Mandya, Dharwad, Mysore, Hassan, Shivamogga, Haveri, and Belgaum; While, Kodagu, and Chikmagalur districts were clustered in IB. Similarly, Cluster II was divided into cluster IIA and cluster IIB. Subcluster IIA comprises Udupi, Gulbarga, Gadag, Raichur, Dakshin Kannad, Uttar Kannad, Koppal and Bellary, and Davanagere district was grouped under cluster IIB.

### Spatial point pattern analysis of RBD

**Figure 4:**
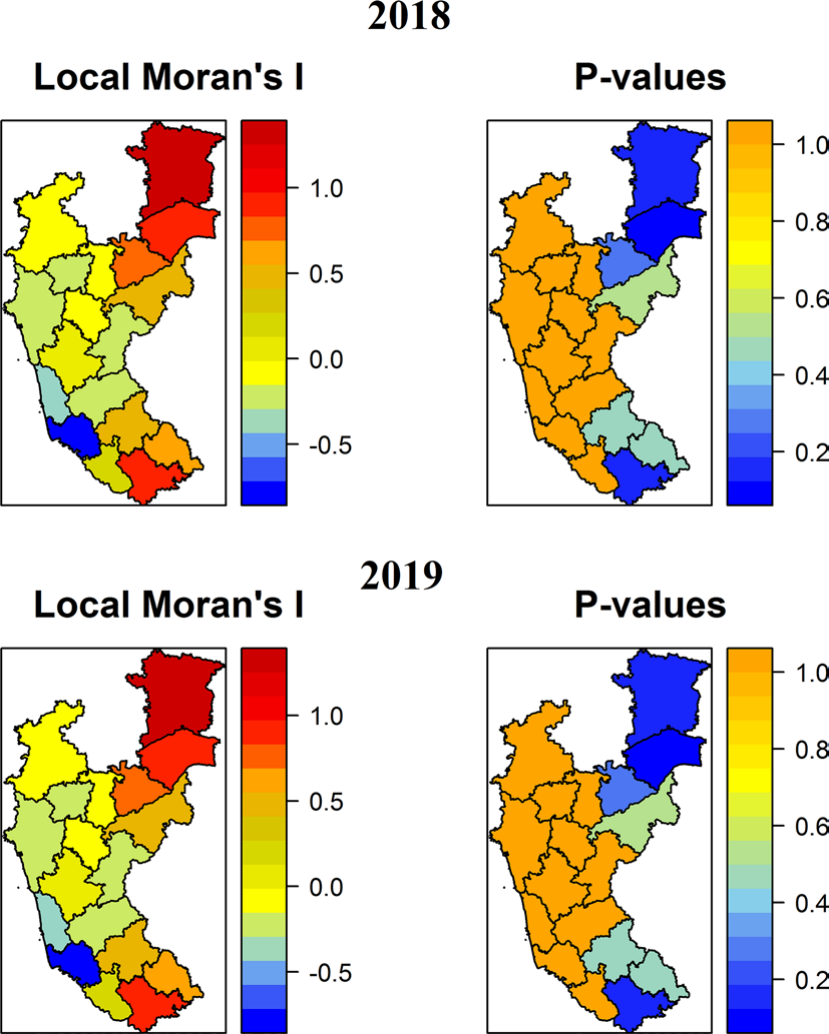
Spatial Point Pattern Analysis of RBD based on Morons I. The statistical significance was observed at two different *p*-values (< 0.1* and < 0.05**). The varied colored areas displayed the dispersed and aggregated clusters of RBD severity during 2018 and 2019. The maps were created using R software (version R-4.0.3).

The cluster and outlier analysis was done using Local Moran’s I and *p*-values. The analyses have identified RBD cluster patterns at the district level during 2018 and 2019, representing dispersed and aggregated clusters of severity (Fig. 4). Based on positive I value, most of the districts were clustered together (at I > 0), except the coastal districts such as Uttar Kannad, Udupi, Dakshin Kannad, and interior districts such as Dharwad, Davanagere, and Chikmagalur, which exhibited negative I value (at I < 0). Similarly, the positive spatial autocorrelation was observed in the districts of Coastal, Hilly, Bhadra, and UKP ecosystems, at higher *p-*values, whereas at lower *p*-values, the districts of TBP and Kaveri ecosystems were clustered together.

**Figure 5:**
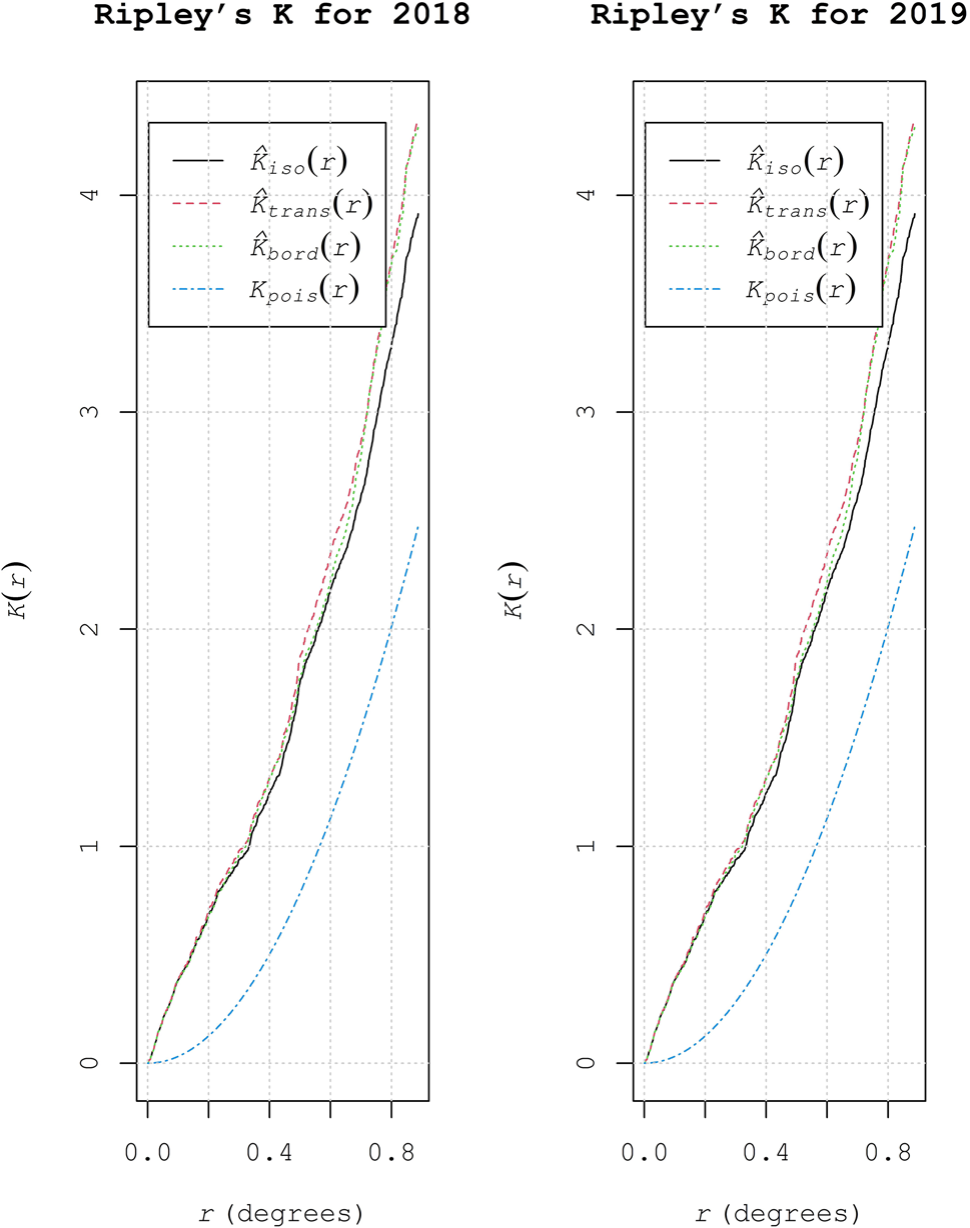
Ripley’s K function values for different sampling sites exhibiting the spatial patterns of RBD in Karnataka during 2018 and 2019.

Further, to characterize the strength of spatial dependence at spatial point pattern analysis, Ripley’s K function was utilized. In both the years of study, statistically significant clustering was observed at larger distances (Fig. 5). Each point under consideration exhibited a greater number of neighbors with increased evaluation distances. The average numbers of neighbors were greater at distances 0.4 and 0.8 representing the significant cluster distribution.

### Surface interpolation to the explicit spatial distribution

#### IDW interpolation approach

**Figure 6:**
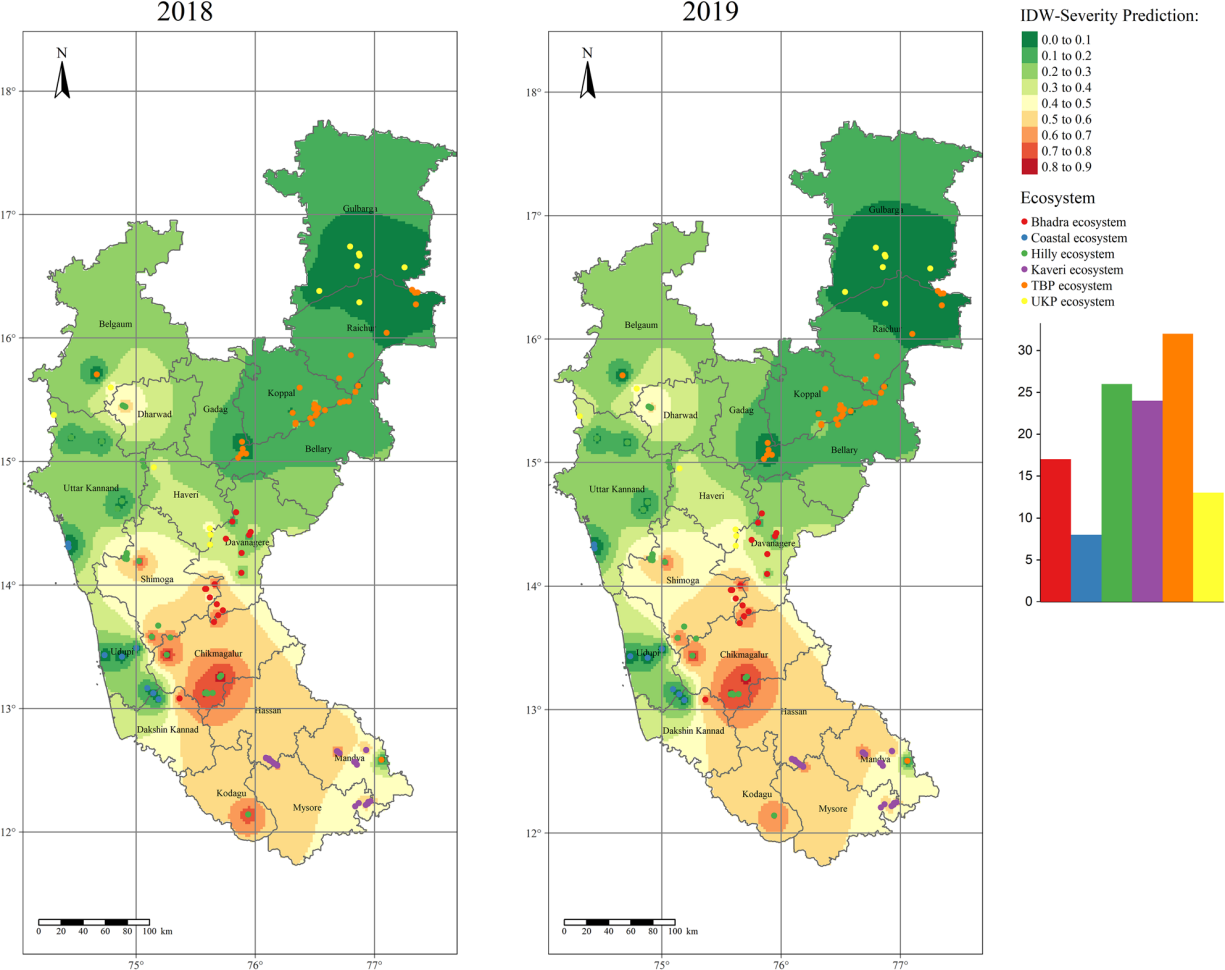
Interpolated disease severity maps of RBD were generated for 2018 and 2019 using the inverse distance weighted tool. Green to Red colors indicate lower to higher disease severity points in different rice ecosystems of Karnataka. The maps were created using R software (version R-4.0.3).

Inverse distance weighted (IDW) interpolation identifies the cell values using a linearly weighted combination of a set of sample points. Contour maps created using the IDW procedure exhibited the RBD distribution pattern in different rice ecosystems of Karnataka (Fig. 6). During both the years of evaluation, the Hilly ecosystem, middle and southern parts of Karnataka has posed a potential risk to RBD with higher disease proportions (> 70%), with focal points at Chikmagalur, Kodagu, and Shivamogga districts followed by Kaveri and Bhadra ecosystems with 50–60 percent severity. Upper Krishna Project (0–10%) and coastal (0–10%) ecosystems were less disease-prone areas for RBD with relatively reduced disease indices. However, the TBP ecosystem had moderate disease severity (20–30%). It is evident from the maps of both years that the disease hot spots are majorly in the middle and Southern Karnataka, and cold spots are in Coastal and Northern Karnataka.

**Figure 7:**
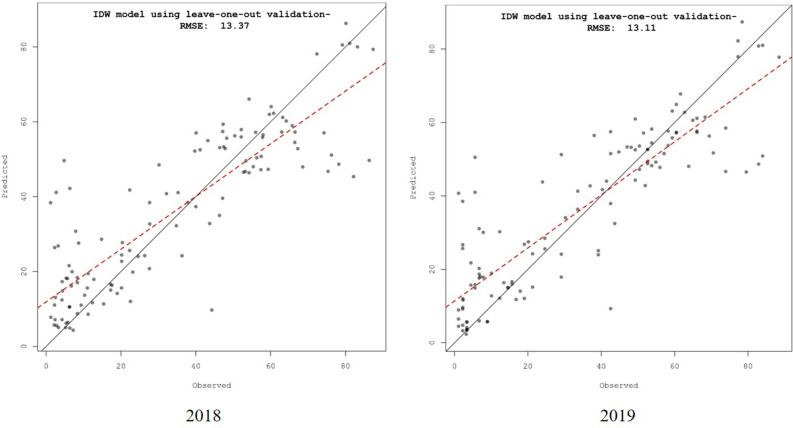
Scatter plot comparing predicted and observed values at the different sampled locations for RBD in Karnataka.

The IDW results were further validated by a scatter plot for predicted severity against observed severity during 2018 and 2019 (Fig. 7). From the plot, the predicted and observed severity almost lies along the line, excluding the errors during both years. The plot values representing the RBD during 2018 and 2019 exhibited a similar severity with RMSE values of 13.37 and 13.11, respectively.

#### Ordinary and indicator kriging

**Figure 8:**
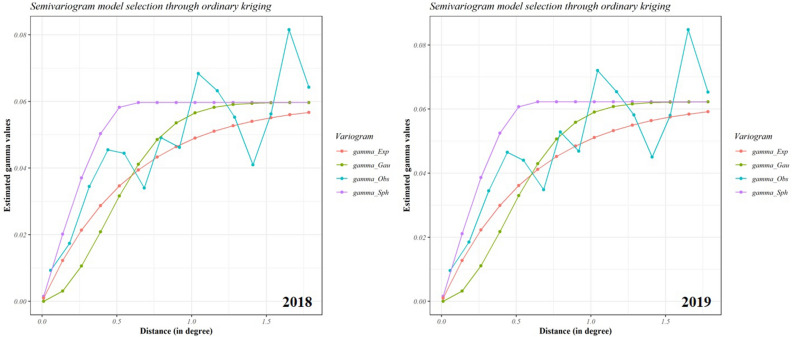
Semivariogram of different experimental models for rice blast disease severity during 2018 and 2019. The colored lines depict the different models such as spherical (purple), exponential (red), and Gaussian (green) models that depict the spatial autocorrelation of measured sample points. Blueline indicates the observed values.

Spatial patterns of RBD severity observations were determined by semivariogram experimental models, such as spherical, exponential, and Gaussian. Among the models, the spherical model was found to be the best fit based on cross-validation of the semivariogram results (Table 2) that exhibited lower mean square error (MSE), root mean square standard error (RMSE), and average standard error (ASE) values (Fig. 8).

**Table 2:** Cross-validation results of semivariogram experimental models on RBD disease severity during 2018 and 2019.

Model	Range (in degree)	Partial sill (C + C_0_)	Nugget (C_0_)	MSE	RMSE	ASE
**2018**						
Spherical	0.59486	599.8945	0.5	693.1113	26.327	0.789
Exponential	0.59486	599.8945	0.5	820.0335	28.6362	1.0869
Gaussian	0.59486	599.8945	0.5	827.03527	29.7437	1.0017
**2019**						
Spherical	0.59486	630.2836	0.5	719.3061	26.8199	0.7957
Exponential	0.59486	630.2836	0.5	828.5236	28.7841	1.0666
Gaussian	0.59486	630.2836	0.5	832.6147	29.9756	1.0374

*MSE* mean square error, *RMSE* root mean square standard error, *ASE* average standard error.

In the spherical model, MSE, RMSE, and ASE values for 2018 were 693.11, 26.327, and 0.789, respectively. The nugget, range (in degrees), and partial sill values were similar in all the models (Table 2). The spherical model was also found fit for the 2019 data with lower MSE (719.3061), RMSE (26.8199), and ASE (0.7957) values.

**Figure 9:**
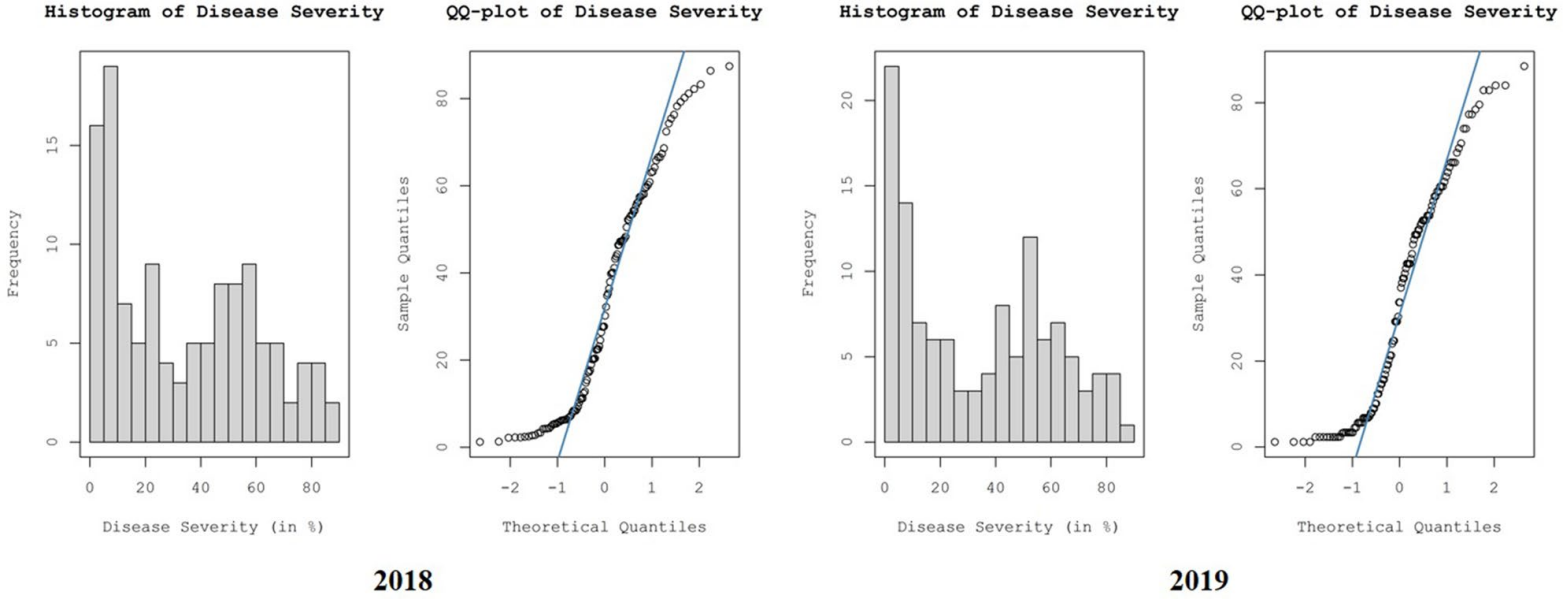
Histograms and normal QQ plots of RBD severity to understand the distribution of the dataset.

RBD severity in different rice ecosystems of Karnataka during 2018 and 2019 followed a normal distribution, as revealed by the Kolmogorov–Smirnov test, which was depicted through histograms and normal QQ plots of the dataset (Fig. 9). Before kriging and interpolation, a slight global trend in the data was removed using the first-order nominal trend removal function.

**Figure 10:**
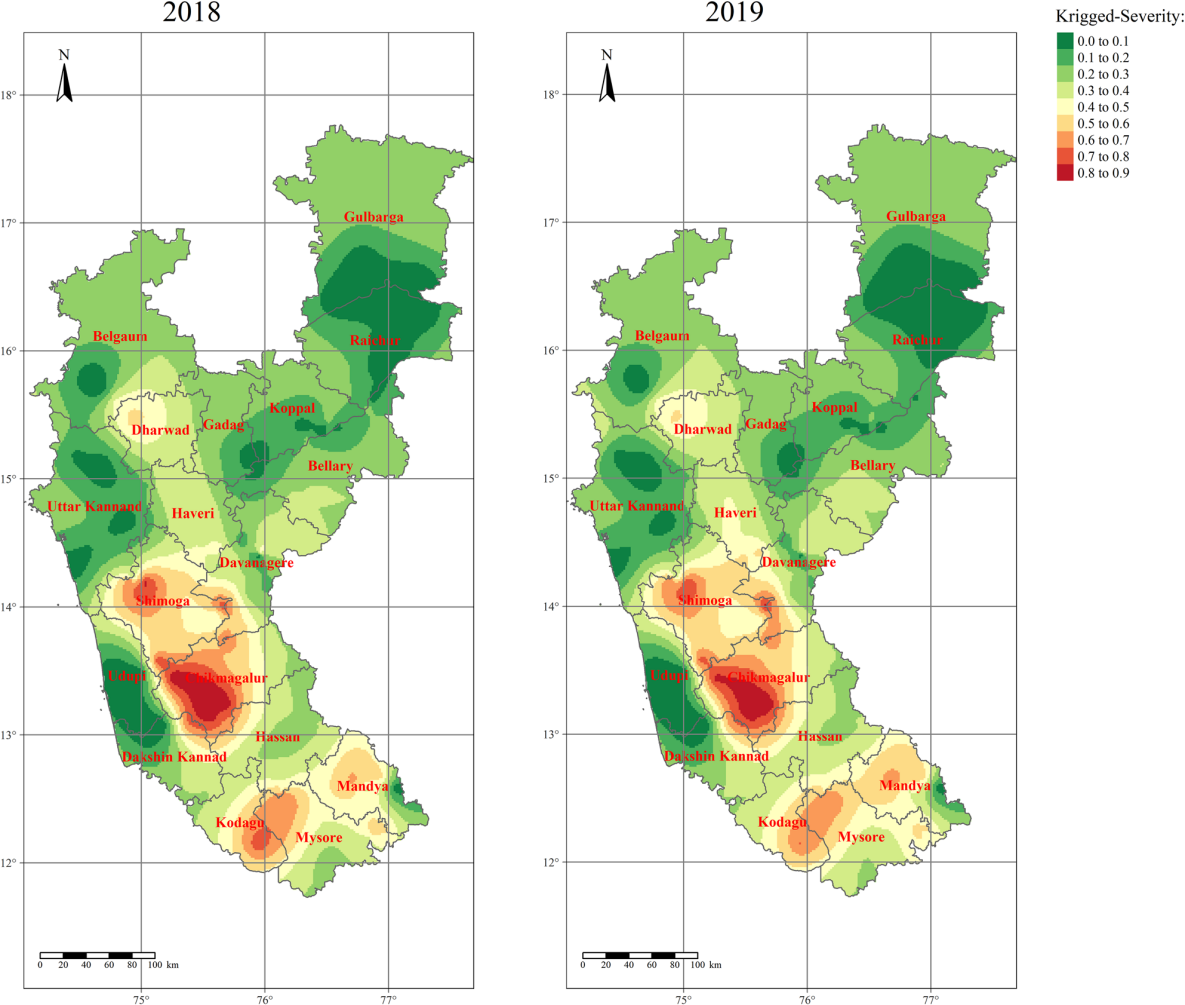
Ordinary kriging interpolated maps representing the spatial distribution of RBD in different rice ecosystems of Karnataka during 2018 and 2019. Green to red-color coded surfaces depicts lower to higher disease severe points. The maps were created using R software (version R-4.0.3).

As with the IDW interpolation technique, ordinary kriging (OK) and indicator kriging (IK) were used to find the spatial surface areas of RBD in different rice ecosystems by considering the severity observations (n = 120). The OK map revealed the maximum severity of RBD in the Chikmagalur, Shivamogga, and Kodagu districts of the Hilly ecosystem with 60–80 per cent severity during 2018 and 2019 (Fig. 10). Districts of Kaveri (Mysore, Mandya, and Hassan), Bhadra (Davangere), Varada (Haveri), and part of the Hilly (Dharwad) ecosystem were found to be with 40–60 per cent severity. At the same time, districts of the Coastal ecosystem and TBP ecosystem exhibited less severity of RBD.

**Figure 11:**
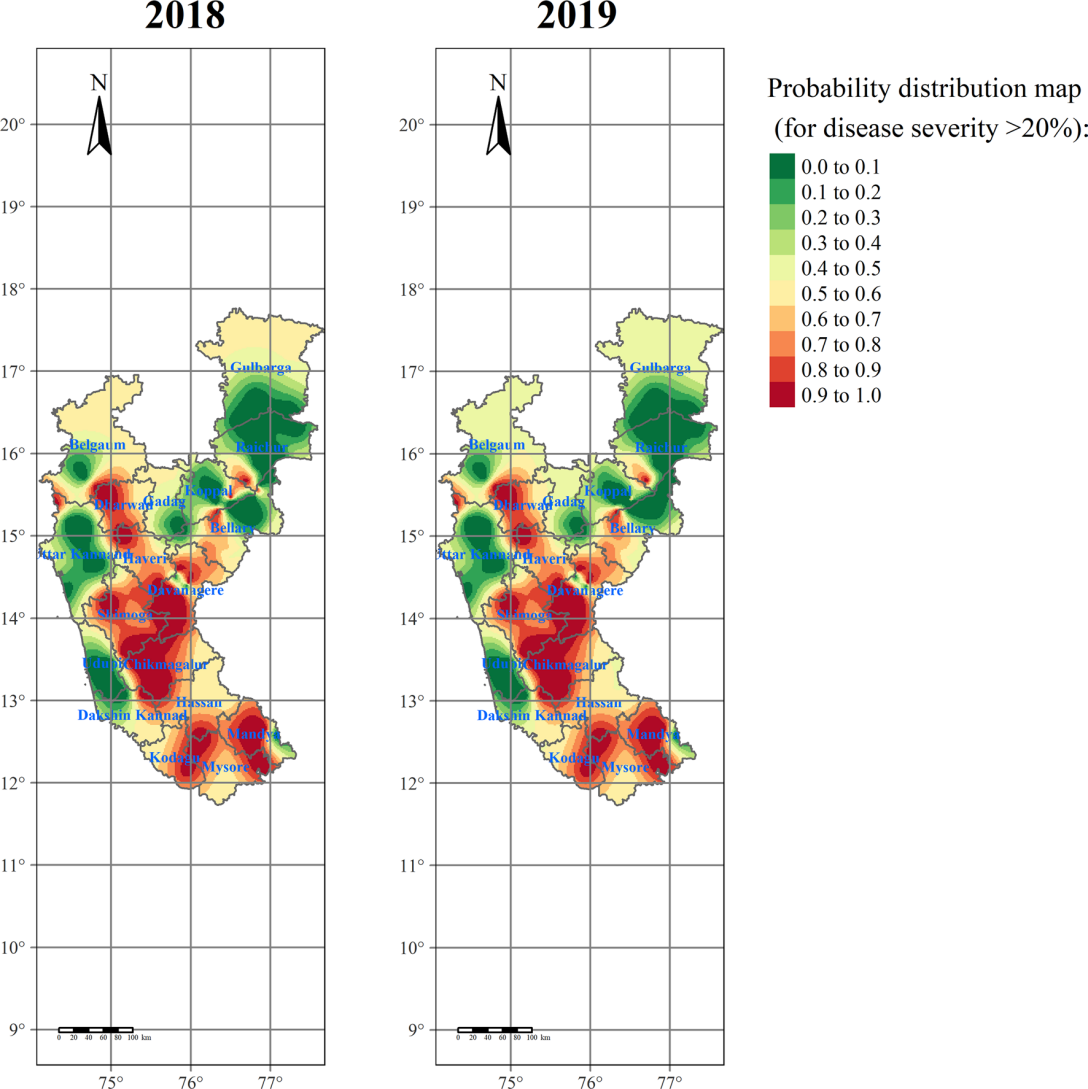
Rice blast disease probability distribution map for Karnataka generated through semivariogram model information using indicator kriging. Green to red-colored points depicts lower to higher levels of risk-prone areas of RBD. The maps were created using R software (version R-4.0.3).

However, in the case of IK, the RBD was more severely distributed (during both 2018 and 2019) around the Hilly (Chikmagalur, Dharwad, Kodagu, and Shivamogga), Bhadra (Chikmagalur, Davanagere, and Shivamogga), Varada (Haveri), and Kaveri (Mandya and Mysore) ecosystems (Fig. 11). Very little distribution was observed in UKP (Belgaum and Gulbarga), TBP (Bellary, Gadag, Koppal, and Raichur), and Coastal ecosystem (Uttar Kannad, Udupi, and Dakshin Kannad). The perusal of results from OK and IK indicated that irrigated ecosystems comprising Hilly, Bhadra, Varada, and Kaveri belts had shown potential risk areas to RBD, and certainly, these areas need utmost attention to reduce and contain further spread to neighboring districts or ecosystems.

## Discussion

Since the rice blast disease (RBD) report in India^30,31^, it has been known to occur in traditional rice-growing ecosystems. However, with increased demand for rice, most of the non-traditional areas shifted towards rice cultivation. A significant proportion of rice produced is lost each year due to RBD^32^. Expansion of cultivable rice areas to these regions has posed a potential risk to RBD over a period. In this context, it was necessary to understand the spatial distribution of RBD in different traditional and non-traditional rice ecosystems of Karnataka. Although the disease status of rice blast in Karnataka was studied in the past, the information on ecosystem wise was lacking. In the present investigation, for the first time in India, the current status and spatial distribution of RBD were identified using geostatistical approaches such as spatial interpolation, autocorrelation, point pattern, and variogram analysis.

The present study identified moderate spatial clusters of RBD by the Local Moran's I spatial autocorrelation (LISA). *M.* *oryzae* produces asexual spores (conidia) during the disease cycle, which reserves disease propagules under field conditions. The conidia of RBD are dispersed by air currents and act as significant determinants of the spread and severity of the disease^33^. The clustering of points in a different ecosystem may also be due to the movement of the pathogen with seed material from one field to another^34^. The movement of the pathogen through air currents in the direction of the wind and the seed materials to shorter and longer distances might be the reason for the generation of clusters in the map.

Point pattern analysis was used to identify the hotspots of RBD in different rice ecosystems of Karnataka. From the analysis, the hot spots were identified in the Hilly ecosystem consisting of Chikmagalur, Shivamogga, Kodagu, and Dharwad districts. These spots need the extensive management strategy of RBD since the disease is known to affect > 70 per cent in these areas. These areas with high rainfall are congenial for RBD pathogen to proliferate and invade. Our findings were supported by a previous report by Suzuki^35^, where he found the influence of topographic factors in the incidence and intensity of RBD. He observed the increased severity of RBD from plains to the foothills and between the mountains. This may be due to congenial conditions for RBD in and around the hilly regions^8^. The less severity of RBD was observed in the TBP ecosystem than the Kaveri ecosystem due to the more application of chemical fertilizers. The farmers of the Kaveri ecosystem apply fewer fungicides to manage RBD, but the farmers of the TBP ecosystem apply excessive fungicides to manage RBD^27^.

In the present study, the percent severity of RBD was considered to generate the spatial distribution maps across the studied areas of Karnataka. Similarly, the data was generated at unsampled points using the surface interpolation tools like inverse distance weighting (IDW), ordinary kriging (OK), and indicator kriging (IK). The RBD semivariogram indicated relatively moderate spatial dependency. IDW is simple and quick; however, kriging is complex and time-consuming but provides the best linear unbiased estimates^36^. Based on the generated spatial clusters in the interpolation tools, the kriging is more accurate than IDW.

The possible processes in the spatial pattern of RBD are the dispersal of the pathogen through the air and the distribution of susceptible/ resistant plant cultivars^37^. Another reason for the spatial distribution of RBD might be the terrain that affects microclimate^38^. The hilly areas with higher altitudes create a characteristic microclimate with lower night temperature, frequent and lengthy dew duration, and reduced sunshine hours. These are congenial conditions for the RBD severity^8^. The cluster size in these conditions can be as large as hilly areas or flat areas lying between hilly areas.

The present study has identified the RBD risk areas in different rice ecosystems of Karnataka. The study shows that the disease hot spots are majorly in the middle and southern parts of Karnataka, and cold spots are in Coastal and Northern Karnataka. The disease-prone areas, viz., hilly, and irrigated ecosystems (Kavri, Bhadra and TBP) require special attention. The disease will be severe in Chikmagalur, Kodagu, and Shivamogga districts of the hilly ecosystem since these areas have congenial weather for the disease development.

The Karnataka state of India has 31 districts, which are divided again based on the climatic conditions into 10 Agro-climatic zones. Among these, rice has been widely cultivated in 18 districts under rainfed and irrigated ecosystems. In the areas with different climatic conditions favoring the disease, the RBD can be managed with ecosystem-specific disease management strategies. The present study is also useful to other nations with similar climatic conditions (such as mid-east countries) in identifying the risk areas to RBD. The information generated in the current study would provide valuable information to the extension personnel in formulating site-specific management strategies against RBD and also to create awareness among the rice growers. The data generated will seek the breeder’s attention in developing ecosystem-specific resistant rice cultivars.

In conclusion, our present study demonstrates that the clustering of RBD spatial patterns has significant implications in deciding management strategies. The aggregated patterns of RBD at a regional scale provide an opportunity to arrange the nursery fields by considering the altitude and weather conditions. The distribution pattern of RBD over time and space will allow the farmers and scientific community to concentrate on the resources such as labor and chemicals within a small area, thereby increasing the efficiency in the site-specific resource management. Professional pest or disease control systems should be promoted in the high-risk hilly areas located at the borders of districts or states. Overall, the presence of considerable RBD clusters or hotspots in different rice ecosystems of Karnataka might help to design the appropriate disease management strategy.

## Materials and methods

### Study area and data collection

The study was carried out by gathering data from 120 sampling sites of 18 administrative districts distributed under five irrigated (Bhadra, Kaveri, Thunga Bhadra, Upper Krishna, and Varada) and two rainfed (Coastal and Hilly) ecosystems of Karnataka during *Kharif* (June to September) of 2018 and 2019, respectively (Fig. 1 and Table 1).

Three fields were selected for the study in each sampling site, and observations were recorded by selecting a hundred plants randomly in each field by walking diagonally. Disease scoring was carried out using the 0–9 scale (Supplementary Table S1) according to Standard Evaluation System (SES) for Rice^39^. The severity of rice blast was expressed as Percent Disease Index (PDI) using the formula (1) as given below^40^.

1 $$\mathrm{PDI}=\frac{\mathrm{Sum\, of\, individual\, ratings }}{\mathrm{No}.\mathrm{ \, of\, leaves\, assessed} \, \times \, \mathrm{Maximum \, disease\, grade \, value}} \times 100$$

### Data pre-processing and validation

The data were processed for the normality using Kolmogorov–Smirnov test^41^. Further histograms and standard QQ plots were computed to understand the data distribution to remove the slight global trend observed in the dataset. The severity of RBD (%) in different rice ecosystems of Karnataka was analyzed using the Kruskal–Wallis test in R software (version R-4.0.3)^42^ to find out the variation in disease severity across studies areas. Agglomerative hierarchical cluster analysis was performed using the average linkage method based on the severity of RBD to infer the distances among the districts^43^. Data optimization and cluster analysis were performed through the 'hclust’ function using R software (version R-4.0.3). In an average linkage hierarchical clustering, the distance (L) between two clusters (r,s) is the distance between two points and can be expressed by formula (2):

2 $$L\left(r,s\right)= \frac{1}{{n}_{r}{n}_{s}}\sum_{i=1}^{{n}_{r}}\sum_{j=1}^{{n}_{s}}D({X}_{ri ,}{X}_{sj})$$

where X and Y are the observations from clusters r and s, respectively.

### Geostatistical analysis

The spatial distribution of RBD in different rice ecosystems of Karnataka was estimated by point pattern analysis and surface interpolation techniques. The clusters of RBD were identified by point-pattern-optimized hotspot analysis and Ripley's K function. Ordinary kriging (OK), indicator kriging (IK), and inverse distance weighting (IDW) approaches were employed to generate the spatial maps and potential risk areas to RBD.

### Point pattern analysis

Spatial autocorrelation was performed using Moran’s *I* or LISA statistics, and results were optimized by considering the nearest sampling locations. LISA indicates the presence of spatial clusters, and the results were inferred using the *p*-value*.* The Moran’s I statistic was computed using Eq. (3) for a real unit *i*.

3 $${I}_{i}={Z}_{i}\sum_{j}^{n}{\mathrm{W}}_{\mathrm{ij}}{\mathrm{Z}}_{\mathrm{j}}$$

where *I* is the statistic for the district *I*; *Z* is the difference between the RBD severity risk at *i* and the mean RBD severity for regions; *W* is the spatial weights matrix.

The particular nearest areas or sampling sites with higher RBD severity values were considered hotspots or risk areas^44^. The clustering pattern was estimated using Ripley’s *K(r)* function^45^ for the model developed in each area. The function is expressed as *K(r)* = *λ − 1E*, where *K(r)* denotes the characteristics of point events over a range of scales; *E(r)* is the expected mean number of points within a distance *r* of randomly chosen points, and *λ* is the RBD severity of the studied sites.

### Spatial interpolation

The values at the unsampled locations were predicted by using the spatial interpolation approach; for instance, the severities of RBD at sites (*X*_*1*_*,* *X*_*2*_*…..* *Xn*) are (*Z*_*1*_*,* *Z*_*2*_*…..* *Z*_*n*_). With the use of spatial interpolation, the Z values can be estimated at new point X. The diseased surface area was estimated by IDW and OK techniques. The IDW at an unsampled site *i* can be expressed as following formula (4):

4 $${{F\left(i\right)= \sum_{i=1}^{m}W}_{i}Z\left({r}_{i}\right)=\frac{\sum_{i=1}^{m}Z({r}_{i})/ \left|r -{r}_{i}\right|}{\sum_{j=1}^{m}1 / {\left|r -{r}_{j}\right|}^{p}}}^{p}$$

where *P* is the parameter; *m* is  a number of neighboring points taken into account at a certain cut-off distance. The interpolated values are compared with the actual values via leaving one-out-cross validation from the omitted point.

Kriging is an interpolation technique used to estimate the spatial correlation of the random function *Z(X*_*0*_*).* The predicted values of variable *Z* at unsampled point *X*_*0*_ are found using formula 5^46^.

5 $$\gamma \left(d\right)= \frac{1}{2}\sum {\left\{\left[\hat{Z} \left({X}_{1}\right)-Z\left({X}_{2}\right)\right]\right\}}^{2}$$

Using the OK technique, the surface maps of the RBD severity were constructed using the following Eq. (6):

6 $$\hat{Z}\left({X}_{0}\right)=\sum_{i=1}^{n}{\lambda }_{i}Z({X}_{i})$$

where *Z* is the variable of interest at spatial coordinates *X*_*i*_ and *X*_*o*_; *n* is the number of neighbors associated with the sampling point; *λ*_*i*_ is the weight associated with sampling point *X*_*i*_ and the *i*th observation point^47^. Semivariograms calculated the closest neighbor index based on the average spatial variability and the RBD severity^48^. The semivariograms were fitted with different models, and the exponential model was found best and used for the generation of OK maps. Semivariogram is defined as following formula (7):

7 $$\hat{y}\left(h\right)=\frac{1}{2N(h)}\sum_{i=1}^{N(h)}{\left[Z\left({X}_{i}\right)-Z({X}_{i}+h)\right]}^{2}$$

where *γ(h)* is the semivariance for the interval distance class *h*, *N(h)* is the number of data pairs of a given lag interval distance and direction, *Z* *(x*_*i*_*)* is the measured sample value at point *i*, and *Z* *(x*_*i*_ + *h)* is the measured sample value at position *I* + *h*.

Semivariogram values are fitted with spherical, exponential, and Gaussian models as:

Spherical model:

8 $$\hat{\rm y}\left(h\right)={C}_{0 }+ C\left[1.5\frac{h}{a}-{\left(\frac{h}{a}\right)}^{3}\right], if 0\le h\le a.$$

Exponential model:

9 $$\hat{y}\left(h\right)={C}_{0 }+ C\left[1-\mathrm{exp}\left\{-\frac{h}{a}\right\}\right] for h\ge 0$$

Gaussian model:

10 $$\hat{\rm y}\left(h\right)={C}_{0 }+ C\left[1-\mathrm{exp}\left\{-\frac{{h}^{2}}{{a}^{2}}\right\}\right] for h\ge 0$$

For the spherical model, *Co* is a nugget, *(C* + *Co)* is sill, and *a* is range. Whereas *a* represents the theoretical range for exponential and Gaussian models.

The accuracy of the estimated data across applied models and methods was critically compared by deriving accuracy measures such as average standard error (ASE), mean square error (MSE), and root mean square error (RMSE). Indicator kriging (IK) was used to find out the disease vulnerable areas where the severity of RBD was more than 20% per field^8,49,50^. Based on this, the probability risk maps were generated by taking account of the best-fitted semivariogram model. A similar method was followed to generate a color-coded map for ordinary kriging where the contour symbolization represents the higher risk areas of RBD in different rice ecosystems of Karnataka.

## Supplementary Information

Supplementary Table S1.

## Data Availability

The data presented in this study are available on request from the corresponding author.
