# Supplementary material for: Spatial distribution and identification of potential risk regions to rice blast disease in different rice ecosystems of Karnataka
Source: Sci Rep. 2022 May 6;12:7403. doi: 10.1038/s41598-022-11453-9 (PMC9076900; doi:10.1038/s41598-022-11453-9)
Supplement: Supplementary file 1 — Supplementary Table S1. [file 41598_2022_11453_MOESM1_ESM.docx]

**Spatial distribution and identification of potential risk regions to rice blast disease in different rice ecosystems of Karnataka**

Chittaragi Amoghavarsha^1,2^, Devanna Pramesh^2^*, Shankarappa Sridhara^3^, Balanagouda Patil^1^, Sandip Shil^4^*, Ganesha R. Naik^1^, Manjunatha K. Naik^1^, Shadi Shokralla^5^, Ahmed M. El-Sabrout^6^, Eman A. Mahmoud^7^, Hosam O. Elansary^8^, Anusha Nayak^2^ and Muthukapalli K Prasannakumar^9^

^1^ Department of Plant Pathology, University of Agricultural and Horticultural Sciences, Shivamogga, Karnataka, India;

^2^ Rice Pathology Laboratory, All India Coordinated Rice Improvement Programme, University of Agricultural Sciences, Raichur, Karnataka, India

^3^ Center for Climate Resilient Agriculture, University of Agricultural and Horticultural Sciences, Shivamogga, Karnataka, India

^4^ Research Centre, Division of Social Sciences, ICAR-Central Plantation Crops Research Institute, Mohitnagar, Jalpaiguri, West Bengal, India

^5^ Centre for Biodiversity Genomics, University of Guelph, Guelph, ON N1G 2W1, Canada; S.S.

^6^ Department of Applied Entomology and Zoology, Faculty of Agriculture (EL-Shatby), Alexandria University, Alexandria 21545

^7^ Department of Food Industries, Faculty of Agriculture, Damietta University, Damietta, Egypt

^8^ Plant Production Department, College of Food & Agriculture Sciences, King Saud University, Riyadh 11451, Saudi Arabia

^9^ Department of Plant Pathology, College of Agriculture, GKVK, University of Agricultural Sciences, Bengaluru, Karnataka, India

* Correspondence: [parmi.iari@gmail.com](mailto:parmi.iari@gmail.com) (D.P) and [sandip.iasri@gmail.com](mailto:sandip.iasri@gmail.com) (SS)

**Supplementary** **Table S1.** Disease scoring scale for rice blast disease used to record disease severity (SES, IRRI, 2013)

| **Score** | **Lesion type** |
| --- | --- |
| 0 | No lesions present |
| 1 | Small pinpoint size brown specks or larger brown specks without sporulating centre |
| 3 | Small, 1-2 mm diameter, roundish to slightly elongated necrotic sporulating spots, with a distinct yellow halo and brownish margin |
| 5 | Slightly elliptical or Narrow lesions, 1-2 mm in breadth, more than 3 mm long with a brown margin |
| 7 | A broad spindle-shaped lesion with a yellow, brown, or purple margin |
| 9 | Rapidly coalescing small, whitish, greyish, or bluish lesions without distinct margins |
